# Surgical Curative Approach for Severe Hypertension in Select Patients With Underrecognized Rare Renal Tumors: A Case Report

**DOI:** 10.1155/criu/8149819

**Published:** 2025-09-15

**Authors:** Noah Kevin McGreal, Zachary Dylan Winnegrad, Gregory Joseph Diorio, Ron Gefen, Ruth Carolina Birbe, Aileen Grace P. Arriola

**Affiliations:** ^1^Cooper Medical School of Rowan University, Camden, New Jersey, USA; ^2^Urology Division, Department of Surgery, Cooper University Health Care, Camden, New Jersey, USA; ^3^Department of Radiology, Cooper University Health Care, Camden, New Jersey, USA; ^4^Department of Pathology, Cooper University Health Care, Camden, New Jersey, USA

## Abstract

Juxtaglomerular cell tumors (JCTs), also known as reninomas, are rare masses with an extremely low risk of malignancy, but their endocrine activity can lead to medication-resistant hypertension and electrolyte imbalances, which may harm patients. Approximately 150 cases have been documented in the literature. In this report, we describe the case of a 40-year-old male with a complex cystic renal mass, prior hemorrhagic strokes, and hypertension who underwent surgical resection. The final pathology confirmed a JCT, marking the first case on record diagnosed at our institution in 20 years. Following surgery, the patient's hypertension improved, and his need for medication decreased. We suggest that physicians managing renal masses that are otherwise suitable for surveillance should include JCT in their differential diagnosis and consider surgical removal if hypertension is present.

## 1. Introduction

Juxtaglomerular cell tumors (JCTs), also known as reninomas, are rare, typically benign growths comprised of specialized smooth muscle juxtaglomerular cells that secrete renin. These cells play a key role in regulating blood pressure by activating the renin–angiotensin–aldosterone system, which increases systemic blood pressure [1, 2]. In cases of JCTs, excessive renin production manifests as unexplained, treatment-refractory hypertension along with electrolyte disturbances and hyperaldosteronism [1, 3, 4]. Patients are typically young adults and often present with a classic triad of headache, hypertension, and hypokalemia, although any or all of these features may be absent [2, 5, 6]. They may also be diagnosed as incidental renal masses due to the increased use of abdominal CT scans [2, 5, 7]. Laboratory tests typically show significantly elevated plasma renin levels, hypokalemia, and hyperaldosteronism [1, 2, 5]. Surgical consultation is recommended if JCT is suspected, as resection typically leads to a cure, except in very rare cases [8].

Here, we report the diagnostic workup and treatment of a 40-year-old hypertensive male patient with a history of multiple hemorrhagic strokes, who was ultimately diagnosed with a renal mass identified as a JCT. This is the first case of its kind recorded at our institution in a 20-year search. The patient presented for urologic consultation with a complex cystic renal lesion found during the workup of hypertension. Surgical removal was decided upon due to the suspicious characteristics of the mass seen on MRI imaging. The diagnosis of JCT was confirmed only after the final pathologic analysis. It was subsequently suggested that the patient's history of severe hypertension could be explained by the tumor's endocrine activity. While a rare occurrence, we hope this case highlights the role of surgical management in curing JCTs and their associated secondary hypertension.

## 2. Case Presentation

**Figure 1:**
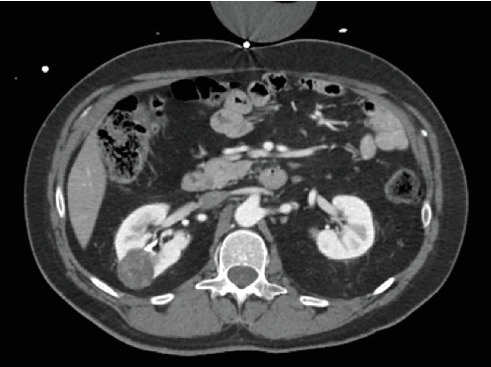
A CT scan of the abdomen and pelvis with IV contrast shows a 3 cm heterogeneous lesion in the right kidney indicative of a complex cystic mass.

**Figure 2:**
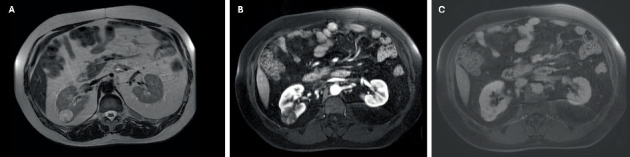
(A) MRI of the abdomen T2 axial image shows internal heterogeneity of the 3.6 cm Bosniak 4 posterior right renal mass more clearly. Axial postcontrast T1 fat-saturated images show (B) early and (C) late enhancements of the mass. Areas of solid enhancement become increasingly visible on late postcontrast imaging.

A 40-year-old male was referred to urology for a right-sided renal mass seen on a renal ultrasound performed during his hypertension workup. The lesion had been previously identified on a CT scan as a heterogeneous 3 cm complex cystic mass following a motor vehicle collision (Figure 1). On follow-up MRI, the lesion was characterized as a Bosniak 4 complex cystic mass, raising concern for renal cell carcinoma (Figure 2). A recommendation for robotic partial nephrectomy was made, and the patient agreed to and consented to the procedure.

His medical history included hypertension diagnosed about 5 years prior that has been refractory to combination therapy, including angiotensin-converting enzyme inhibitor, aldosterone receptor blocker, and vasodilator. The patient also had a history of multiple hemorrhagic strokes, resulting in residual weakness. He was followed by nephrology, who had ordered laboratory workup for the hypertension. Serum creatinine, electrolytes, and 24-h urine metanephrines and catecholamines were all within normal limits. Both serum renin and aldosterone levels were significantly elevated, which at the time was attributed to the typical causes of hypertension. An ultrasound and MRI were ordered by nephrology, which led to a urologic consultation and consideration of a possible JCT.

**Figure 3:**
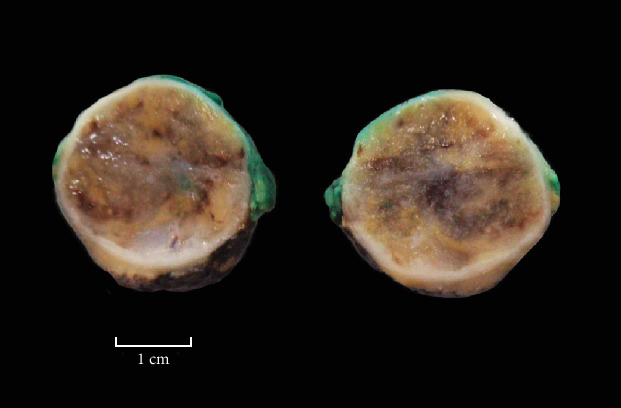
Gross pathology of right renal mass partial nephrectomy, green ink at parenchymal margin.

**Figure 4:**
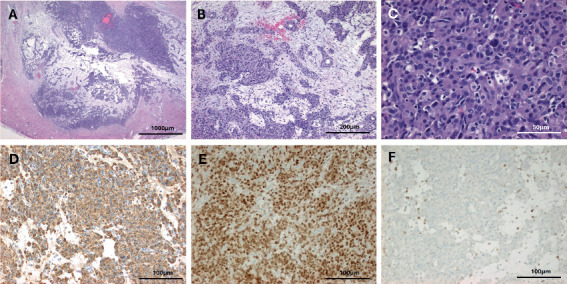
Histopathology and immunohistochemical (IHC) stains of the juxtaglomerular cell tumor. (A, B) Encapsulated tumor with prominent vessels (H&E at 20×, scale bar 1000 *μ*m, and 100×, scale bar 200 *μ*m). (C) High-power view showing polygonal to spindle cells with mild pleomorphism (H&E at 400×, scale bar 50 *μ*m). (D–F) IHC for (D) renin and (E) GATA3 is positive, while (F) PAX8 is negative (200×, scale bar 100 *μ*m).

A right robotic partial nephrectomy was performed in standard fashion without complication. Grossly, the tumor measured 3.6 × 3 × 2.7 cm and had a thick capsule, and the cut surface revealed yellow-tan soft tissue that was focally hemorrhagic and vaguely gelatinous (Figure 3). Microscopically, it consisted of a vascular-rich mesenchymal tumor composed of sheets of polygonal to spindle cells with indistinct cell borders, hyperchromatic nuclei, and mild pleomorphism. Centrally, it was edematous, hypocellular with lymphocytes, plasma cells, and scant blood (Figure 4). Immunohistochemical stains were positive for GATA3, caldesmon, CD34, and vimentin while negative for PAX8, AE1/AE3, CK-OSCAR, CD117, HMB45, Melan-A, chromogranin, INSM1, synaptophysin (focal weak staining), desmin, smooth muscle actin, and S100. Beta-catenin showed cytoplasmic and membranous staining. Confirmatory renin immunohistochemical stain performed at a research laboratory was positive, supporting the diagnosis of JCT (Figure 4).

The patient had an uneventful recovery and was discharged on postoperative Day 3. Plasma renin activity returned to normal range several weeks after surgery. Two months postop, the patient was fully healed from a surgical perspective, but home blood pressure readings remained elevated. It was later discovered that he had inadvertently stopped all antihypertensive medications due to a misunderstanding. He was instructed to continue taking only lisinopril and to monitor his blood pressure at home, with follow-up appointments scheduled over the next few months. At the 11-month follow-up, the patient remained normotensive on lisinopril alone and showed no signs of tumor recurrence.

## 3. Discussion

JCTs are rare entities first described in 1967 [9, 10], characterized by excessive renin production and silent detrimental endocrine abnormalities with hypertension, hypokalemia, and secondary hyperaldosteronism [1–4]. Some cases can present with hypertension and normal potassium or nonfunctional tumors [2]. Patients are generally young adults, with ages at diagnosis ranging from 6 to 72 years, and a female predominance [1, 2, 5]. Although this may not represent the true incidence, JCTs are more commonly detected in the second and third decades of life for several reasons. One reason is that new-onset hypertension in this age group often leads to investigations for secondary causes, rather than assuming essential hypertension [1]. Additionally, the increased use of abdominal CT scans has contributed to a higher rate of incidental renal mass detection [2, 5, 7].

When JCTs are diagnosed promptly and treated appropriately, one can achieve an extirpative cure. If overlooked, this could result in poorly managed hypertension and its associated complications, including end-organ damage, retinopathy, neuropathy, and stroke [5]. Diagnosis requires a high level of suspicion, which likely contributes to the overall reported rarity of JCTs [1, 7]. These adverse events are preventable, and with that goal in mind, we aim to raise awareness of JCT within the differential diagnosis of today's physicians. Here, we report the workup and treatment for a hypertensive patient with a history of multiple hemorrhagic strokes and a renal mass diagnosed ultimately as a JCT.

This 40-year-old male patient initially presented with refractory hypertension in the setting of adverse cardiovascular events at an abnormally early onset. Laboratory analysis revealed elevated plasma renin activity and aldosterone simultaneously, a characteristic finding of JCT. Subsequent imaging led to the identification of a Bosniak 4, 3.6 cm complex cystic mass, which alone is justification to offer a partial nephrectomy. Had this been a smaller renal lesion, it could have easily been monitored with active surveillance, and hypertension likely would have persisted [11]. Evaluation of a patient at urologic consultation for renal masses should purposefully focus on the presence of hypertension, hypokalemia, or hyperaldosteronism to raise suspicion for JCT [1, 2, 5].

Ultimately, histopathology remains the gold standard for confirming the diagnosis of JCTs [2]. Other entities, such as Wilms tumor, renal cell carcinoma, and renal oncocytoma, can also elevate renin levels and appear as complex renal masses on imaging [6]. Several features help distinguish the diagnosis, such as the presence of rhomboid renin protogranules in the cytoplasm and abundant endoplasmic reticulum in JCT cells when examined under electron microscopy [2, 12–14]. JCTs will also exhibit a distinct vascular growth pattern described as a hemangiopericytoma-like pattern [2, 12]. Additional genomic tumor analyses can further aid in diagnosis. JCTs have been shown to exhibit loss of chromosomes 9 and 11, gains of chromosomes 3, 4, 10, 13, 17, and 18, and upregulation of over 400 genes including renin and CD117 [2, 15, 16]. Together, these studies enable an accurate diagnosis of JCTs, highlighting the critical role of the pathologist.

When diagnosed early and surgically removed, the prognosis is excellent [1, 2, 5]. After surgical resection, over 90% of patients with JCT maintain normotensive blood pressure and no longer require antihypertensive medications [1, 2, 5, 7]. Since nearly all JCTs are benign, complete resection is typically curative, with an exceptionally low recurrence rate. A nephron-sparing approach is preferred when feasible, which usually involves laparoscopic partial nephrectomy. However, radiofrequency ablation has also proven effective, although it has less supporting follow-up data [17, 18]. Conversely, should the diagnosis elude the physician, outcomes can be catastrophic. There have been reports of malignant behavior with JCT metastasizing to the lungs [2, 19] and massive terminal brain hemorrhage in a patient with long-standing undiagnosed JCT [20]. Pathologic features that may suggest malignancy in JCT include vascular invasion and large tumor size [2]. As with any form of hypertension, the longer it goes unchecked, the more detrimental downstream effects can occur. We emphasize the importance of including JCT in the differential diagnosis for renal masses in hypertensive patients and strongly advocate for their curative removal.

## Data Availability

The data that support the findings of this study are available on request from the corresponding author. The data are not publicly available due to privacy or ethical restrictions.
